# Clinical Epidemiology Features and Risk Factors for Acute Diarrhea Caused by Rotavirus A in Vietnamese Children

**DOI:** 10.1155/2023/4628858

**Published:** 2023-06-27

**Authors:** Dang Van Chuc, Dang Phuong Linh, Dang Viet Linh, Pham Van Linh

**Affiliations:** ^1^Hai Phong University of Medicine and Pharmacy, Hai Phong City, Vietnam; ^2^Hai Phong Children's Hospital, Hai Phong City, Vietnam

## Abstract

**Introduction:**

Acute diarrhea caused by group A rotavirus (RVA) is a leading cause of morbidity and mortality globally in children less than 5 years old. Acute diarrhea caused by RVA is often manifested by loose/watery stool leading to different degrees of dehydration. The detection of risk factors, diagnosis, and prompt treatment of acute diarrhea caused by RVA is critical. We aimed to describe clinical epidemiological features of acute diarrhea caused by RVA and its associated risk factors. *Subjects and Method*. We conducted a cross-sectional study that included 321 children under 5 years old with acute diarrhea at Haiphong Children's Hospital, Vietnam, from 1 August 2019 to 31 July 2020.

**Results:**

Among the 321 children included in our analysis, 221 (68.8%) children were positive for RVA. Males represented 61.1% of cases, 41.2% of children were in the 12-<24-month age group, and the majority of cases were among children in suburban areas (71.5%). Clinical manifestations included loose and watery stool (100%), vomiting-fever-loose/watery stool (57.9%), vomiting-loose/watery stool (83.2%), fever-loose/watery stool (58.8%), dehydration (30%), hyponatremia (22.1%), hypernatremia (1.4%), and hypokalemia (15%). Risk factors for acute diarrhea caused by RVA included history of diarrhea, not exclusive breastfeeding in the first 6 months, living area, maternal education, and income.

**Conclusions:**

Acute diarrhea due to RVA was very prevalent in children under 5 years old. Clinical manifestations included a high prevalence of loose/watery stools/day and dehydration with electrolyte disorder. Mothers should exclusively breastfeed their children for the first 6 months to avoid the risk of acute diarrhea caused by RVA.

## 1. Introduction

Acute diarrhea is defined as loose/watery stool at least 3 times within 24 hours and typically lasting from 5 to 14 days [1–4]. Clinical symptoms and signs of acute diarrhea caused by group A rotavirus (RVA) include vomiting, fever, loose/watery stool, and dehydration [5–8].

To date, seven groups of rotavirus (group A-G) have been isolated and characterized. Group A rotavirus, the most common rotavirus, causes more than 90% of all infections in humans [9]. Rotavirus is transmitted primarily and directly from person to person by the fecal-oral route [10].

Group A rotavirus is the leading cause of acute diarrhea and hospitalization in children under 5 years of age [11–16]. The incidence of acute diarrhea due to RVA fluctuates from 12.7% to 55.0% [5, 8, 11–17] depending on different geographic regions and the possibility of detecting RVA; for example, in Eastern Mediterranean regions, acute diarrhea ranges from 19% to 78.2% [18]. Acute diarrhea caused by RVA affects children's physical development, causes economic burden for the family and society, and is a leading cause of mortality in children under 5 years of age [18–23].

There are several known risk factors for RVA which include number of vomiting episodes a day, not being exclusively breastfed in the first 6 months of life, a history of recent acute diarrhea, sex, malnutrition, living area, maternal education, maternal income, maternal age, and maternal profession. Understanding clinical symptoms and signs, including subclinical signs of acute diarrhea due to RVA, and its risk factors from children and from their mother is very important for doctors in order to provide appropriate treatment to reduce severe complications and reduce overall morbidity and mortality.

In order to more efficiently detect RVA, quick test for detecting the antigen of RVA in acute diarrhea in children under 5 years old has been implemented in routine practice recently in Haiphong Children's Hospital. This study is aimed at describing clinical epidemiologic features of acute diarrhea caused by RVA and its risk factors in children under 5 years old. We hope that the results will help pediatricians promptly diagnose and treat acute diarrhea caused by rotavirus and advise mothers based on risk factors to reduce the likelihood of having this disease in their children.

## 2. Materials and Methods

The cross-sectional study was done from August 1, 2019, to July 31, 2020. The study site was Haiphong Children's Hospital, Vietnam.

### 2.1. Study Population

The study was carried out on 321 children with acute diarrhea of which 221 children had quick test for detecting the antigen for RVA.

### 2.2. Inclusion Criteria

Patients who were enrolled in the study had to meet the following criteria [1–4]:

- Loose/watery stool at least 3 times within 24 hours, lasting no longer than 14 days
- A positive fecal sample for RVA on admission

### 2.3. Exclusion Criteria

Exclusion criteria are as follows: acute diarrhea with blood in the stool lasting longer than 14 days, or not providing informed consent for participation in the study.

### 2.4. Patient Assessment

On admission, patient had a standardized collection which included a fecal sample to test for RVA, an electrolyte test, assessment of clinical signs and symptoms, and associated and risk factors. Group A rotavirus was detected by using Onsite® Rotavirus Ag Rapid Test of CTK Biotech, Inc., USA. Patients who were RVA-positive (case) were used to study clinical epidemiologic features, and patients who were RVA-negative (control) were used to evaluate risk factors.

### 2.5. Data Analysis

SPSS software version 22.0 (SPSS Inc., Chicago, IL, USA) was used to enter and analyze data. Chi-square test was performed to compare 2 percentages; statistical significance was defined with a *p* value less than 0.05.

Odds ratios (OR) with 95% CI were calculated to assess the relationship of acute diarrhea due to RVA and different risk factors.

### 2.6. Ethical Approval

Approval for the study was obtained from the Medical Ethic Council of Haiphong University of Medicine and Pharmacy, and informed consent was obtained according to the Declaration of Helsinki.

## 3. Results

Table 1 shows that among the 321 patients with acute diarrhea, 221 (68.8%) patients had a positive RVA rapid test and 100 patients (31.2%) had a negative RVA rapid test. Those who were RVA-positive were 61.1% male, 27.6% were less than 12 months of age, 41.2% were 12-<24 months of age, 19.4% were 24-<36 months of age, and 5.9% were 36-<48 months of age and 48-<60 months of age, respectively. 71.5% of the patients were from suburban areas, while 26.7% were from urban, and 1.8% were from the island.

**Figure 1:**
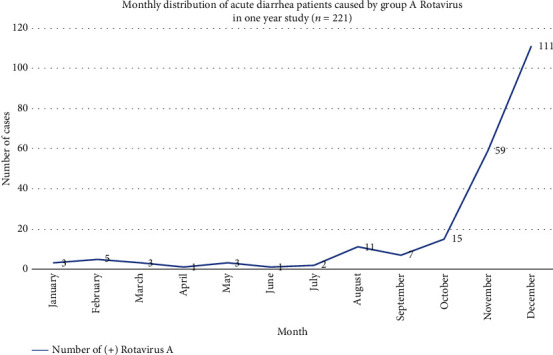
Monthly distribution of acute diarrhea patients caused by group A rotavirus in a one-year study (*n* = 221).

Figure 1 shows that the majority of the patients were surveyed between August and December 2019 with large decreases in patients from January to July 2020. The number of patients seen in November 2019 was 59 (26.7%), and in December 2019, the number increased to 111 (50.2%). From January to July 2020, the number of patients ranged from approximately 1 to 5 cases a month.

Table 2 shows different symptoms; on admission, the majority presented with loose/watery stool with 195 (88.2%) reporting this symptom; 35 (15.8%) had vomiting, and 9 (0.9%) had abdominal pain.

Table 3 shows different clinical feature of the patients; 100% had loose/water stool; 83.2% of the patients had vomiting and watery/loose stool; 58.8% of the patients had fever-loose/watery stool; 57.9% had vomiting-fever-loose/watery stool; and 30% had mild dehydration. We did not note any case of severe dehydration.

Other symptoms included hyponatremia in 49 (22.1%) of the patients, 3 (1.4%) had hypernatremia, 33 (15.0%) had hypokalemia, 13 (5.9%) had hyperkalemia, 19 (8.6%) had a reduction of blood chlorine, and 49 (22.2%) had an increase in blood chlorine (see Table 4).

Table 5 shows that among the 7 reported risk factors, 4 risk factors were statistically associated with acute diarrhea due to RVA. These included the number of times vomiting each day, breastfeeding exclusively in the first 6 months, seasonality, and having a history of diarrhea in the last 2 weeks. Among the 5 maternal risk factors, 3 risk factors were statistically associated with acute diarrhea due to RVA and included living area, maternal education level, and maternal income (see Table 6).

## 4. Discussion

### 4.1. Clinical Epidemiologic Features

Viral infections are the leading cause of acute diarrhea in children under 5 years old globally, and RVA is the most common cause and reason for hospitalization in this age group [24]. Our study showed that 68.8% patients with acute diarrhea were diagnosed with RVA. This incidence was higher than that of Thompson et al. [25] in Ho Chi Minh City (46.8%), Prasetyo et al. [26] in Bandung Indonesia (44.8%), and Nakawesi et al. [27] in Uganda (45.4%). The reason for our high incidence was likely due to patients being hospitalized predominately from August to December 2019 which coincided with cold and dry months in Vietnam. During this period, it is more likely that acute diarrhea in patients was a result of RVA.

In contrast, from January to July 2020, a period which coincided with hot months in Vietnam, acute diarrhea was more likely caused by infections not related to RVA, and we saw a significant reduction in patients hospitalized during this period (Figure 1). In our sample, acute diarrhea due to RVA was more concentrated among males (61.1%), those in suburban area (71.5%), and patients who were 12-<24 months of age (41.2%). These results were consistent with the results of a study from Nguyen et al. [28] that documented approximately 49.6% males and 57.6% in the 12-<24-month age group.

Acute diarrhea due to RVA being more common in males and children in suburban areas is likely a result of gender imbalance and larger suburban populations in Vietnam. According to Mosisa et al. [21], the likely explanation for the high risk among those in the 12-<24-month age group might be that this age group is undergoing complementary feeding, which may cause them to be more vulnerable to diarrheal disease-causing infectious agents due to their underdeveloped immunity. Moreover, children at these ages are starting to crawl and walk and may pick up dirty or contaminated objects and put them in their mouth. The 2016 EDHS report revealed that diarrhea prevalence was high among those in the 12-<24-month age group due to weaning and walking that often occur during these ages, contributing to increased risk of contamination from the environment [29]. In our patients, acute diarrhea due to RVA occurred all year but increased significantly in cold and dry months (from 11 cases in August 2019 to 111 cases in December 2020).

From January to July 2020, Haiphong, Vietnam, suffered from the COVID-19 epidemic, and as a result, people had to isolate at home, so the number of patients coming to the hospital was sporadic. This significantly impacted the monthly distribution of acute diarrhea due to RVA in 2020. In China, according to Zhao et al. [8], the highest number of patients were seen in October (54.8%) and the lowest number of patients were seen in July (5%). August to October are cold and dry months creating conditions for RVA to multiply and cause acute diarrhea.

Approximately 90% of our patients were admitted to the hospital because of loose/watery stool. However, some patients also reported abdominal pain, and nearly 16% of the patients reported vomiting. These were common manifestations seen in acute diarrhea due to RVA (see Table 2). We also found that 100% of the patients had loose/watery stool, 83.2% had loose/watery stool combined with vomiting, 58.8% had loose/watery stool combined with fever, and 57.9% had loose/watery stool combined with vomiting and fever (see Table 3). These results were consistent with that of Nguyen et al. [28]. We did not have any cases of severe dehydration, while only 30% of the patients were moderately dehydrated, and the remainder were not dehydrated. These results may be explained by mothers giving their children low osmotic oral rehydration solution before admitting the hospital.

We noted that among our patients, 100% were tested for electrolytes, mainly due to dehydration. Having all patients undergo electrolyte testing done may be considered to be a waste of testing costs. We also note that nearly 6% of the patients had mild hyperkalemia which could be explained by the patient's acute diarrhea with acidosis or/and by the rupture of red blood cell during the blood test (see Table 4).

### 4.2. Some Risk Factors of Acute Diarrhea due to RVA

Among the 7 risk factors assessed, 4 risk factors were associated with acute diarrhea due to RVA. These risk factors were cold and dry season, number of vomiting ≥5 times a day, not exclusive breastfeeding in the first 6 months, and history of acute diarrhea (see Table 5). The cold and dry season risk factor finding was consistent with the other study done in Vietnam [28], Mozambique (June-July: coldest and drier months) [30], China [31], Thailand [32], Korea [33], and Japan [34]. Our findings of patients vomiting ≥5 times a day were similar to Li et al. [35] who found that patients with acute diarrhea due to RVA reported vomiting ≥3 times a day (OR = 8.788). Breast milk especially colostrum contains many factors against the infections of digestive tract due to both bacteria and RVA such as IgA, white blood cell, and antibacterial peptides, so breastfed child can reduce the risk of acute diarrhea in general and acute diarrhea due to RVA in particular. This comment was consistent with the comment of Black and Armstrong [36] and D Tino De Franco et al. [37]. Our results showed that children who were not fully breastfed during the first 6 months had an AOR of 3.98 and 95% CI from 2.02 to 7.48 and a *p* value less than 0.05.

Rotavirus infection could happen several times in one's life from neonate to elderly [38] due to the weak and unstable immunity following rotavirus infections. This study indicated that a child having history of acute diarrhea had 2.39 times increased risk of recurrence compared with a child without this history. Similarly, Gladstone et al. [39] found that the protective efficacy of previous *rotavirus infection* against the next moderate and severe infection was 3 times lower than that of other infections.

Among the 5 maternal factors, 3 factors were statistically associated with acute diarrhea caused by RVA. These included patients living in suburban area (OR = 2.16), low maternal education level (OR = 4.72), and low maternal income (OR = 6.25). Our results were consistent with that Nakawesi et al. [27], Xuan [40], Thiam et al. [41], and Claudine et al. [17].

## 5. Conclusions

Acute diarrhea caused by RVA was common in pediatric practices with manifestation such as loose/watery stool and dehydration, often seen in cold and dry season. In this study, associated factors such as seasonality, vomiting ≥5 times a day, not exclusive breastfeeding in the first 6 months, history of acute diarrhea, living area, low maternal education level, and low maternal income were risk factors of acute diarrhea caused by group A rotavirus.

## 6. Limitations

Because most of the study period of 2020 coincided with the outbreak of COVID-19 in the city, it partly affected the number of patients who came to the hospital for acute diarrhea and may also affect some conclusions drawn from the statistics particularly risk factors.

## Figures and Tables

**Table 1:** Epidemiological features of children with acute diarrhea caused by group A rotavirus.

Variables	Number of patients	Percentage (%)
Group A rotavirus		
Positive	221	68.8
Negative	100	31.2
Sex (*n* = 221)		
Boy	135	61.1
Girl	86	38.9
Age (month) (*n* = 221)		
<12	61	27.6
12-<24	91	41.2
24-<36	43	19.4
36-<48	13	5.9
48-<60	13	5.9
Areas (*n* = 221)		
Urban	59	26.7
Suburban	158	71.5
Island	4	1.8

**Table 2:** The admission reason of children with acute diarrhea caused by rotavirus (*n* = 221).

Admission reasons	Number of patients	Percentage (%)
Abdominal pain	8	0.9
Loose and watery stool	195	88.2
Vomiting	35	15.8

**Table 3:** The clinical features of children with acute diarrhea caused by rotavirus (*n* = 221).

Symptoms and signs	Number of patients	Percentage (%)
Loose/watery stool	221	100
Vomiting, fever, and loose/watery stool	128	57.9
Vomiting and loose/watery stool	184	83.2
Fever and loose/watery stool	130	58.8
No dehydration	154	69.7
Some moderate dehydration	67	30.03
Severe dehydration	0	0.0

**Table 4:** Electrolyte (*n* = 221).

Indices		Number of patients	Percentage (%)	Normal range
Na^+^ (mmol/L)	<135	49	22.1	135-145 mmol/L
	135-145	169	76.5	
	>145	3	1.4	

K^+^ (mmol/L)	<3.5	33	15.0	3.5-4.5 mmol/L
	3.5-4.5	175	79.1	
	>4.5	13	5.9	

Cl^−^ (mmol/L)	<100	19	8.6	90-110 mmol/L
	100-110	153	69.2	
	>110	49	22.2	

**Table 5:** The risk factors of acute diarrhea caused by rotavirus from children (*n* = 321).

Risk factor	Rotavirus (A)				Total	OR 95% CI *p*
	(+)		(-)			
	*n*	%	*n*	%		
Sex						
Boy	135	68.5	62	31.5	197	0.96 0.59-1.56 >0.05
Girl	86	69.4	38	30.6	124	
Age group						
<24 (months)	152	65.2	81	34.8	233	0.52 0.29-0.92 <0.05
≥24 (months)	69	78.4	19	21.6	88	
Season						
Winter-spring	195	74.4	67	25.6	262	3.69 2.06-6.62 <0.05
Summer-fall	26	44.1	33	55.9	59	
Number of vomiting a day						
≥5	86	70.2	16	31.8	102	3.34 1.84-6.09 <0.05
<5	135	61.6	84	38.4	219	
Exclusive breastfeeding in the first six months						
No	115	76.7	35	32.3	150	3.89 2.02-7.48 <0.05
Yes	88	69.8	38	30.2	126	
Malnutrition						
Yes	20	64.5	11	35.5	31	0.81 0.37-1.75 >0.05
No	201	69.3	89	30.7	290	
History of diarrhea in the last two weeks						
Yes	38	17.2	8	8.0	46	2.39 1.07-5.33 <0.05
No	183	82.8	92	92.0	275	

**Table 6:** The maternal risk factors of acute diarrhea caused by rotavirus (*n* = 321).

Risk factors		Rotavirus (A)				Total	OR 95% CI *p*
		(+)		(-)			
		*n*	%	*n*	%		
Area	Urban	61	80.3	15	19.7	76	2.16 1.56-4.03 <0.05
	Suburban/island	160	65.3	85	34.7	245	

Maternal age (year)	<30	144	67.3	70	32.7	214	0.27 0.03-2.3 >0.05
	30-<40	71	71.0	29	29.0	100	0.4 0.04-3.5 >0.05
	≥40	6	85.7	1	14.3	7	1

Maternal profession	Farmers	7	53.8	6	46.2	13	1
	Workers	6	85.7	1	14.3	7	5.14 0.47-55.64 >0.05
	Civil officials	77	70.6	32	29.4	109	2.06 0.64-6.62 >0.05
	Housewives	21	75.0	7	25.0	28	2.57 0.64-10.28 >0.05
	Small business/freelance	97	65.5	51	34.5	148	1.63 0.52-5.1 >0.05

Maternal education level	≤ high school	44	89.8	5	10.2	49	4.72 1.8-12.3 <0.05
	College and higher	177	65.1	95	34.9	272	

Maternal income	Low	25	92.6	2	7.4	27	6.25 1.45-26.9 <0.05
	≥ average	196	66.7	98	33.3	294	

## Data Availability

Data and material used and/or analyzed during the current research are available from the corresponding authors on reasonable request.

## Abbreviations

RVA: Group A rotavirus.
